# Supplementary material for: Polymerase-inhibitor drug synergy and mutational signatures in different epithelial cell models of RSVA and hPIV3 infection
Source: NPJ Antimicrob Resist. 2026 May 29;4:55. doi: 10.1038/s44259-026-00222-7 (PMC13530243; doi:10.1038/s44259-026-00222-7)
Supplement: Supplementary file 1 [file 44259_2026_222_MOESM1_ESM.pdf]

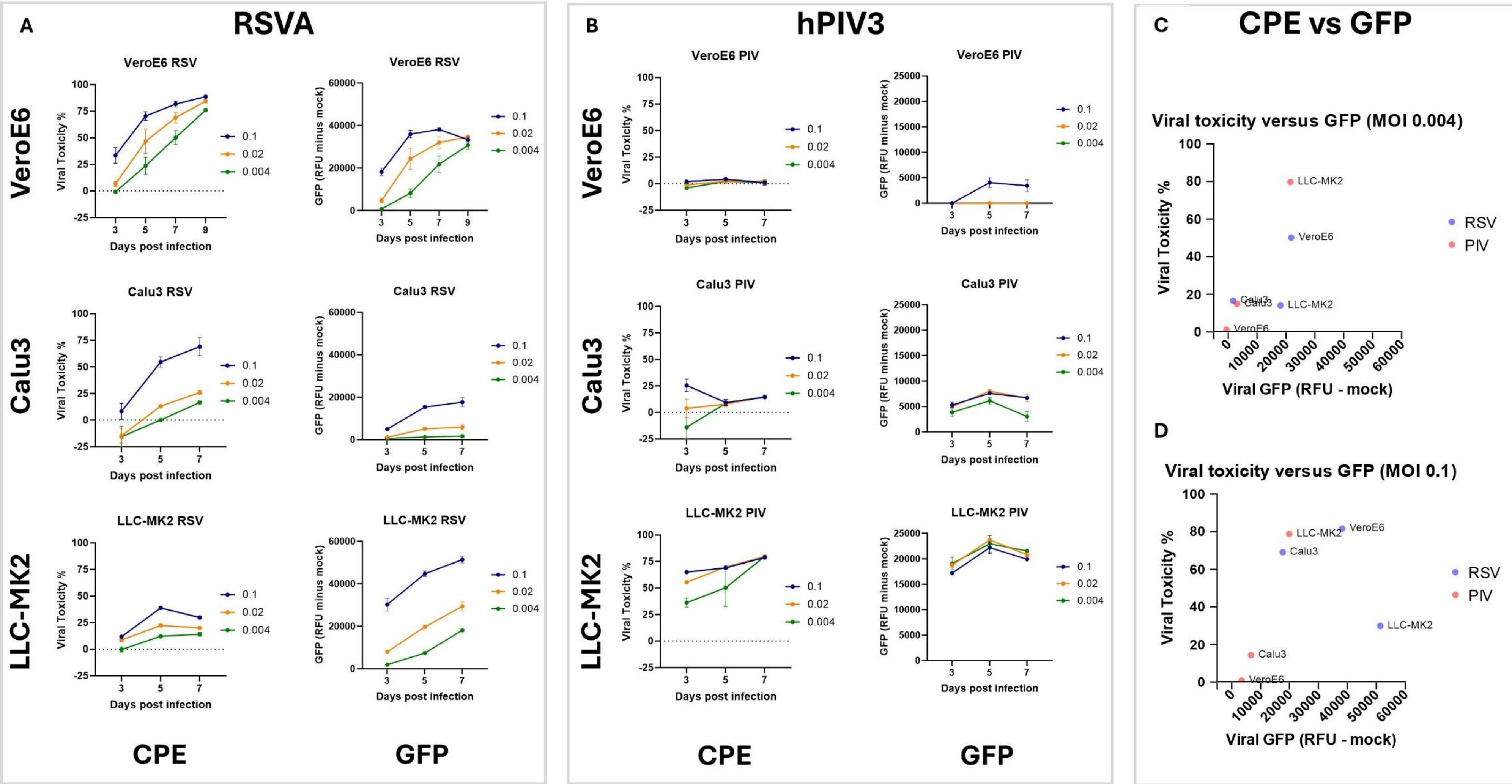

Supplementary Figure 1 Determining optimal epithelial cell line for RSVA and hPIV3 infection assays. A-B**.** Three epithelial cell lines VeroE6, Calu3 and LLC-MK2 were screened for the development of quantifiable infection markers with RSVA (A) and hPIV3 (B). For each pairing of virus and cell type, infections were performed at multiplicity of infections (MOIs) of 0.1, 0.02 or 0.004. Infection progression was determined by either cytopathic effects (CPE) using quantification of endpoint crystal violet staining of surviving cells at each timepoint, or from fluorescence intensity measurements of viral-GFP signal. Measurements were made at timepoints of 3-, 5- or 7-days post-infection. C-D. The extent of CPE and viral-GFP was compared for each cell and virus pairing at a 7 days post-infection endpoint at either an MOI of 0.004 (C) or 0.1 (D). n=3.

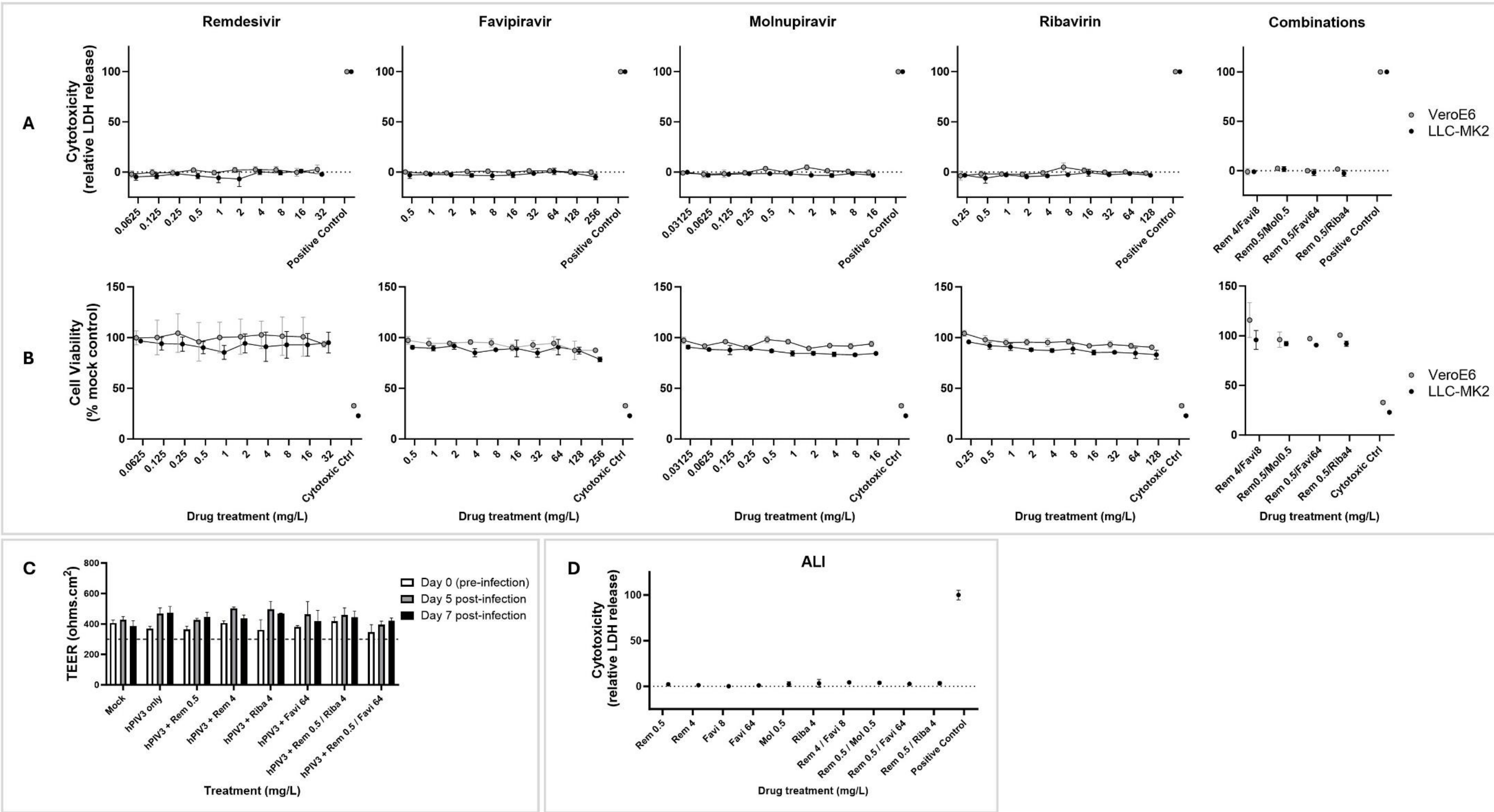

Supplementary Figure 2 Drug cytotoxicity assays for cell lines and primary airway ALI culture. A. VeroE6 and LLC-MK2 epithelial cell lines were exposed to antivirals for 24 hours before quantification of lactate dehydrogenase (LDH) release in culture supernatant, normalised against spontaneous LDH in mock control and maximum LDH activity using a lysis buffer positive control (n=3). B. Cell viability was determined for VeroE6 and LLC-MK2 cells exposed to the experimental antiviral concentrations for 24 hours, using chemical reduction of alamarBlue (n=3). Viability percentage was normalised against mock controls. The cytotoxic positive control condition was 50% DMSO. C. Transepithelial electrical resistance (TEER) was measured for ALI cultures prior to infection (Day 0) and at 5 and 7 days post-infection with hPIV3, with or without addition of antiviral drugs. Values are mean (±SEM) TEER (ohms.cm^2) for 3 independent culture wells. Dashed line at 300 ohms.cm^2 represents expected threshold for intact barrier function for differentiated ALI cultures. D. ALI culture wells (paediatric donor cells on high-throughput 96-well Corning plates 7369) were exposed to antivirals for 24 hours in basolateral media, before quantification of lactate dehydrogenase (LDH) release, as normalised against spontaneous LDH in mock control and LDH positive control (2 independent cultures per condition, triplicate technical assay replicates). Favi = favipiravir, Rem = remdesivir, Mol = EIDD-1931 (molnupiravir), Riba = ribavirin.

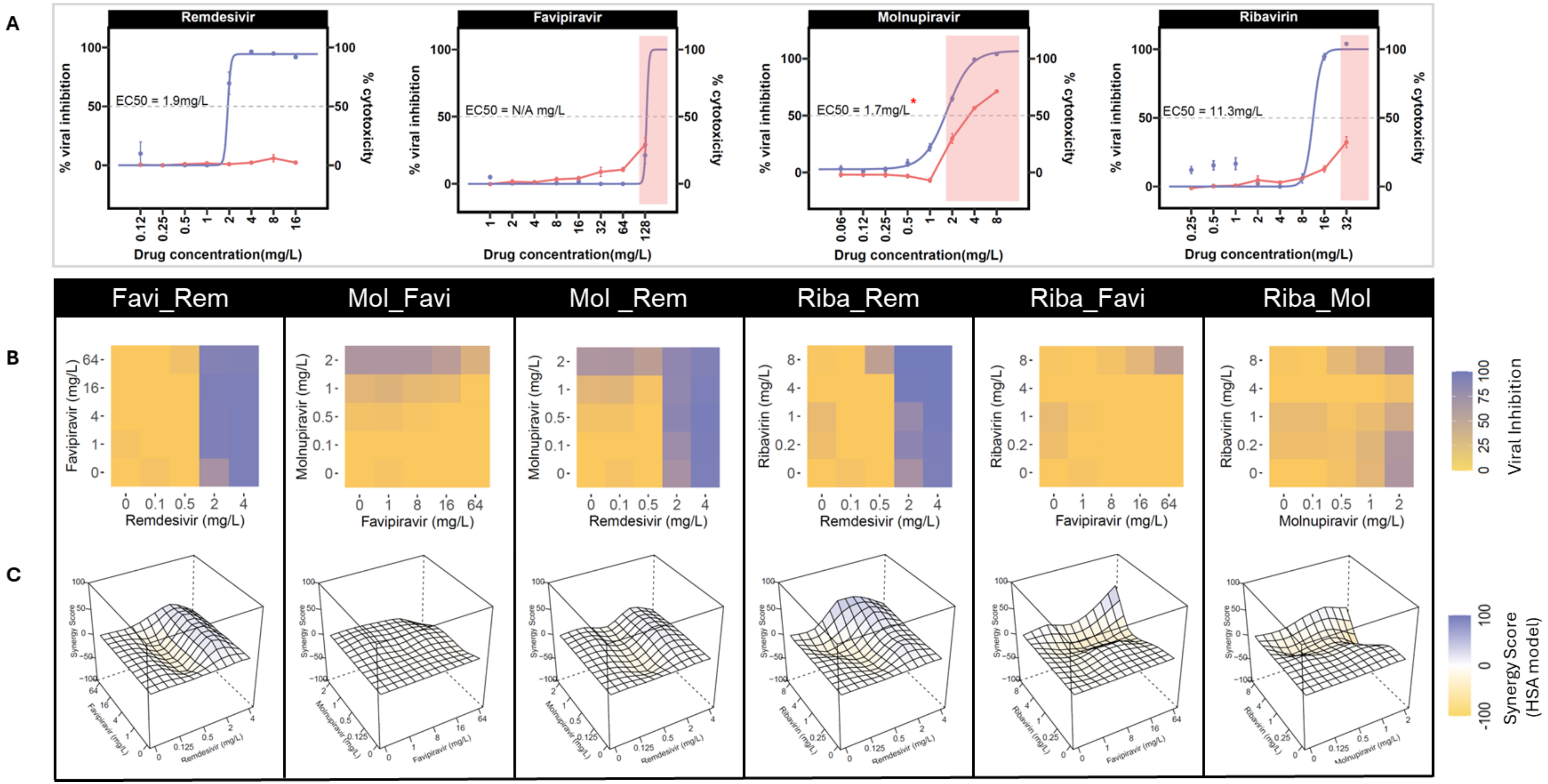

Supplementary Figure 3 Antiviral efficacy modelling using viral-GFP fluorescence intensity. A. Dose response curves for monotherapy concentrations of remdesivir, favipiravir, molnupiravir (EIDD-1931), and ribavirin tested against GFP-hPIV3 infection of LLC-MK2 cells, as modelled by relative reduction in GFP fluorescence intensity at 7 days post-infection). The viral inhibition % model is shown as the blue line, while drug-only endpoint cytotoxicity (as a % reduction of adherent cells) is indicated by the red line. The shaded box indicates drug concentrations with cytotoxicity exceeding 20%. Red asterisk indicates the predicted EC_{50} for molnupiravir from the fitted curve is uncertain, as reduction in relative viral-GFP intensity is related to the drug-only cytotoxicity not viral inhibition. B. The Viral-GFP data was used to calculate hPIV3 inhibitory dose response grids for dual-combinations of antivirals. C. 3D contour plots of synergy scores for the antiviral dual-combinations, as calculated by the HSA model. Favi = favipiravir, Rem = remdesivir, Mol = EIDD-1931 (molnupiravir), Riba = ribavirin. n=3-4 per condition.

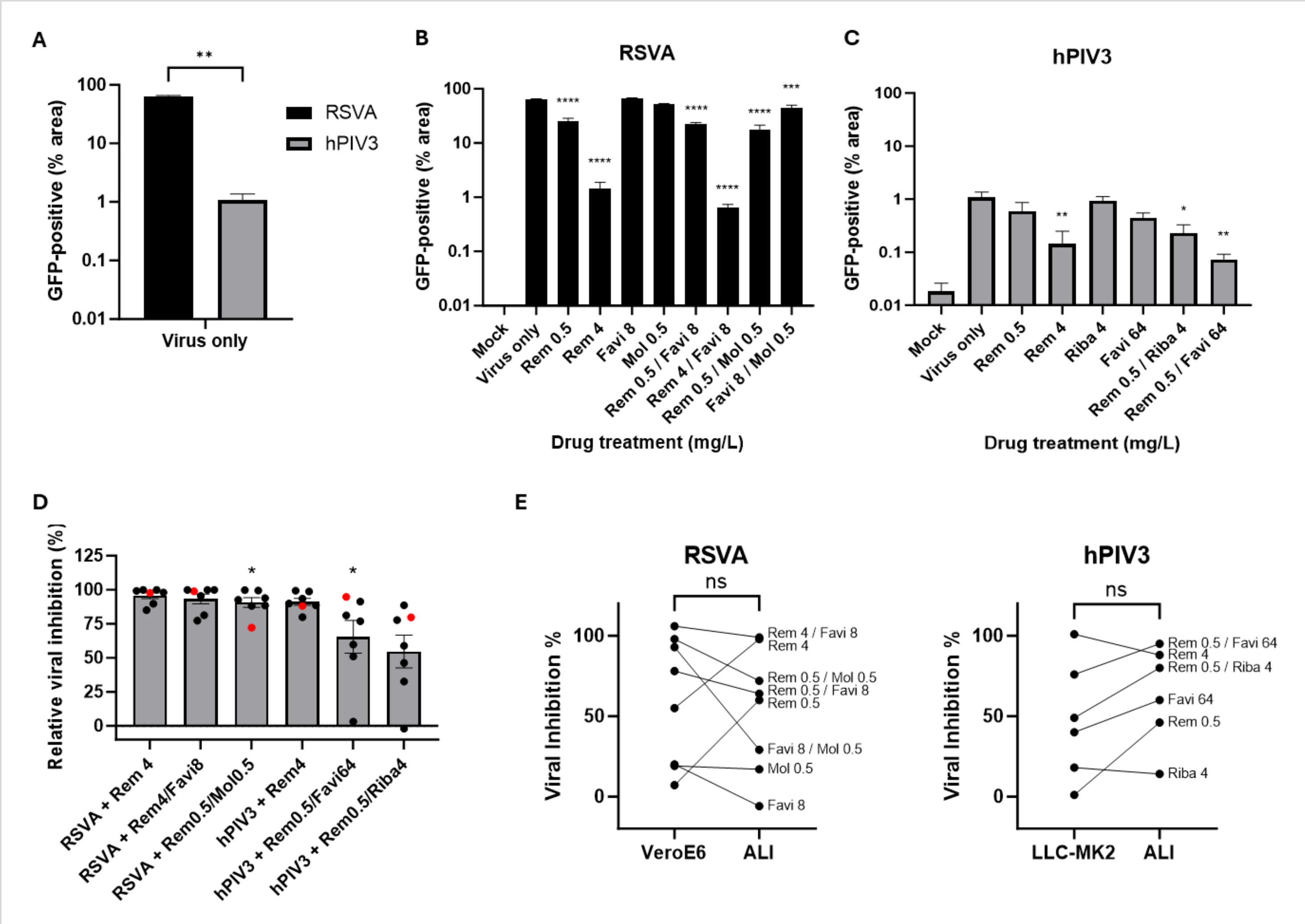

Supplementary Figure 4 Viral GFP quantification for primary airway culture infections. Fluorescence micrograph image analysis was used to calculate the percentage area of each well with positive GFP fluorescence, as a quantification of viral infection. A. Comparison of virus only condition for RSVA and hPIV3. B-C. Quantification of viral GFP with and without antiviral drugs for RSVA (B) and hPIV3 (C), with asterisks indicating conditions with a significant reduction versus virus only control. D. Primary airway cultures were generated as High-Throughput Screening 96-well ALI (using Corning plates 7369) with cells from 6 healthy paediatric donors and infected with RSVA or hPIV3 with same methodology as 24-well ALI model, with or without selected antiviral conditions. Each black data point is the mean relative viral inhibition per donor in the 96-well model, with the red data point the previous result for the 24-well model. Asterisks indicate conditions where result significantly differs between models (by Wilcoxon test). E. Comparison of viral inhibition for matching antiviral concentrations between the respective cell line and ALI models for RSVA and hPIV3 (Wilcoxon matched-pairs test). All figures show values as mean (±SEM) of triplicate ALI culture wells. Favi = favipiravir, Rem = remdesivir, Mol = EIDD-1931 (molnupiravir), Riba = ribavirin. * p<0.05, ** p<0.01, *** p<0.001, **** p<0.0001.
